# Video lectures versus live lectures: competing or complementary?

**DOI:** 10.1080/10872981.2019.1574522

**Published:** 2019-02-06

**Authors:** Lasith Ranasinghe, Lazarie Wright

**Affiliations:** Department of Medicine, Faculty of Medicine, Imperial College London, United Kingdom; College of Medical and Dental Sciences, University of Birmingham, United Kingdom

**Dear Editor**,

We read, with great interest, the article by Brockfeld *et al*. [1] evaluating the use of video versus live lectures. Being medical students at the University of Birmingham and Imperial College London, we have personal experience of the advantages and disadvantages of the two approaches as our universities employ both. The tech-savvy nature of the current millennial generation of medical students coupled with improvements in telecommunication technology opens new avenues for more efficient and effective delivery of medical education. Nonetheless, such educational approaches should not be blindly embraced without careful consideration of potential consequences.

Davis *et al*. (2009) [2] explored students’ perception of a lecture capture programme at the University of Leeds. The most popular uses were for revision or recap of difficult topics, and students appreciated being able to access material in their own time. This highlights the main benefits of video lectures, namely the flexibility and convenience that they provide. Brockfeld [1], however, used video lectures for the initial delivery of information and held the sessions in groups with time constraints. Whilst they demonstrated that both video and live lectures are equally effective at transfer of information, they appear to have neglected the main benefits of video lectures. Furthermore, the study appears to view video and live lectures as competing approaches when, in fact, their relationship could be symbiotic. Scheduled live lectures with video recordings available for revision would be a more efficient and student-friendly approach.

On the contrary, access to video lectures may breed complacency with regard to managing the academic workload. The availability of video lectures relies on the student’s impetus to get through the material in their own time. A lack of motivation to work at a sufficient pace may result in the accumulation of an insurmountable workload before impending exams. Therefore, the benefit of video lectures is likely to be heavily influenced by the attitudes of the individual. This was demonstrated by Howland and Moore (2010) [3] who explored student perceptions of internet-based courses and found that students reporting positive attitudes towards this approach were constructivist learners and demonstrated good self-direction.

Live lectures may provide a more structured learning schedule and promote sustainable management of a student’s workload as the rate of delivery of information is controlled by the medical school. The balance between the use of video and live lectures could be shifted according to the maturity of the students. It is, perhaps, likely that younger students would benefit more from live lectures due to inexperience with managing their workload independently. Older students, on the other hand, may prefer more independence and control of their workload as they must often juggle hospital placements and lectures. Furthermore, the type of information being delivered should also influence this balance. Some topics (e.g., biochemistry) are predominantly delivered via didactic lecture-based transfer of information, which is achieved effectively by video lectures [1]. On the other hand, discussion-based topics (e.g., ethics, clinical cases) and practical skills are conveyed less effectively by video lectures [4].

To conclude, live lectures promote a sustainable work rate and encourage engagement whilst video lectures are a convenient revision tool allowing students to work at their own pace. As such, video lectures should be viewed as a supplement to live lectures rather than a replacement. To optimise the benefit to students, medical schools should also shift the emphasis on either video or live lectures based on the maturity of the students and the type of information being delivered.
